# Age, sex, and the vascular contributors to cerebral pulsatility and pulsatile damping

**DOI:** 10.1152/japplphysiol.00500.2020

**Published:** 2020-09-17

**Authors:** Wesley K. Lefferts, Jacob P. DeBlois, Jacqueline A. Augustine, Allison P. Keller, Kevin S. Heffernan

**Affiliations:** ^1^Department of Exercise Science, Syracuse University, Syracuse, New York; ^2^Department of Medicine, University of Illinois at Chicago, Chicago, Illinois; ^3^Department of Kinesiology, SUNY Cortland, Cortland, New York; ^4^Department of Kinesiology, Iowa State University, Ames, Iowa

**Keywords:** aging, artery stiffness, pulsatile damping, pulsatility, sex differences, wave reflections

## Abstract

Cerebral pulsatility reflects a balance between the transmission and damping of pulsatility in the cerebrovasculature. Females experience greater cerebral pulsatility with aging, which may have implications for sex differences in stroke risk and cognitive decline. This study sought to explore vascular contributors to cerebral pulsatility and pulsatile damping in men and women. Adults (*n* = 282, 53% female) underwent measurements of cerebral (middle cerebral artery) pulsatility, pulsatile damping (ratio of cerebral to carotid pulsatility), large artery stiffening (ratio of aortic to carotid pulse wave velocity), and carotid wave transmission/reflection dynamics using wave intensity analysis. Multiple regression revealed that older age, female sex, greater large artery stiffening, higher carotid pulse pressure, and greater forward wave energy was associated with increased cerebral pulsatility (adjusted R^2^ = 0.44, *P* < 0.05). Contributors to decreased cerebral pulsatile damping included older age, female sex, and lower wave reflection index (adjusted R^2^ = 0.51, *P* < 0.05). Our data link greater large artery stiffening, carotid pulse pressure, and forward wave energy to greater cerebral pulsatility, while greater carotid wave reflection may enhance cerebral pulsatile damping. Lower cerebral pulsatile damping among females may contribute to greater age-associated cerebral pulsatile burden compared with males.

**NEW & NOTEWORTHY** Cerebral pulsatility contributes to brain health and depends on a balance between transmission and damping of pulsatile hemodynamics into the cerebrovasculature. Our data indicate that cerebral pulsatility increases with age, female sex, extracranial artery stiffening, forward wave energy, and pulse pressure, whereas pulsatile damping decreases with age and female sex and increases with greater carotid wave reflections. These novel data identify pulsatile damping as a potential contributor to sex differences in cerebral pulsatile burden.

## INTRODUCTION

Cerebrovascular and cognitive diseases are a substantial burden on the aging population, (12, 18) with cost to exceed $1 trillion a year by 2050 (18). While both sexes are impacted by these diseases, females may experience more severe cerebrovascular disease (4, 42, 48) and greater risk and severity of cognitive disease than males (23). Our understanding of the physiological mechanisms underlying the vulnerability to, or resilience against, developing these chronic cerebrovascular diseases in males and females is limited. Increasing evidence indicates a key role of large extracranial artery function in the development of cerebrovascular/cognitive disease (12, 44, 51). Specifically, the pulsatile manner in which blood flow is delivered to the brain has emerged as a critical contributor to brain health that is dependent on large extracranial artery stiffness (26).

Blood flow generated by the heart is pulsatile, defined by repeated cycles of discontinuous flow. Large elastic extracranial arteries (i.e., aorta and carotid arteries) help convert pulsatile flow generated by the heart to more continuous (nonpulsatile) blood flow to peripheral target organs. Cerebral pulsatility increases with age (48, 57), owing to stiffening (i.e., loss of elasticity) of large extracranial arteries that widens pulse pressure and enhances pulsatile energy transmission into the brain (31). This increase in systemic (28, 43, 48) and cerebral pulsatility (48, 56) appears greater in females compared with males, particularly at older age. Pulsatile cerebral blood flow is a contributor to the pathogenesis of cerebrovascular disease (9, 22, 36, 53) and cognitive impairment (7) and may therefore play a role in observed sex differences in cerebrovascular (4, 42, 48) and cognitive disease prevalence (23). Sex differences in cerebral pulsatility may not solely reflect differences in the transmission of cerebral pulsatility through stiffened extracranial arteries but may also stem from altered defenses against cerebral pulsatility. Indeed, not all individuals may exhibit negative effects of increased artery stiffening and pulsatility (57), suggesting that the brain may have mechanisms through which it can damp and defend against pulsatility with aging.

Pulsatile damping is an emerging physiological concept, separate from pulsatile transmission, which describes the ability of the cerebrovasculature to attenuate blood flow pulsatility as it is transmitted to target organs (57). Pulsatile damping is impaired in older compared with young adults (57) and may further deteriorate in persons with cognitive disease (37). Thus, in addition to increased transmission of pulsatile energy, aging may simultaneously impair the ability to damp and defend against cerebral pulsatility. Whether cerebral pulsatile damping differs by sex and contributes to the greater pulsatile burden observed in females has not been investigated (48, 56). Moreover, the mechanisms behind pulsatile damping are largely unknown and may differ between sexes; however this is currently underexplored. As such, there is a need to identify the vascular contributors to pulsatile damping across the lifespan and whether cerebral pulsatile damping differs by sex.

Based on the current literature there are several potential factors that may influence cerebral pulsatility and pulsatile damping (see Supplemental Table S1; all supplemental material is available at: https://doi.org/10.6084/m9.figshare.12943217), including vascular structure (wall thickness, diameter [28, 40)], vessel wall properties [stiffness (26)], and regional hemodynamics [pulse pressure, energy wave dynamics (21, 26)]. Therefore, the purpose of this study was to examine vascular contributors to 1) cerebral pulsatility and 2) cerebral pulsatile damping from the common carotid to middle cerebral artery (MCA) in males and females across the aging spectrum. Based on current literature it was hypothesized that 1) age and sex would be significant contributors to cerebral pulsatility and pulsatile damping, with older age and female sex conferring greater pulsatility and less pulsatile damping, and 2) extracranial artery stiffness, forward wave energy, and carotid pulse pressure would predict greater cerebral pulsatility, whereas reflected compression wave and forward expansion wave would predict greater pulsatile damping.

## METHODS

### Participants

This analysis used data from 282 adults between the ages of 18 and 85 yr (54% female; Supplemental Table S2) compiled from data collected between 2013 and 2018 in the Human Performance Laboratory at Syracuse University. All participants were generally healthy volunteers from the local Syracuse University and Syracuse community. Exclusion criteria included self-reported smoking, stroke, dementia, diabetes mellitus, previous cardiovascular events, pulmonary/renal/neurological disease, depression, or recent head trauma (concussion). Hypertensive, hyperlipidemic, overweight (BMI 25–30 kg/m^2^), and obese (BMI 30–35 kg/m^2^) individuals were *not* excluded, due to the high prevalence of these risk factors within middle-aged and older adults. Menopausal status (pre-, peri-, postmenopausal) was documented according to STRAW+10 guidelines (15). All participants provided written informed consent before study initiation, and all procedures were approved by the Syracuse University Institutional Review Board and conformed to the standards outlined in the Declaration of Helsinki.

**Table 1.** Descriptive characteristics in young/premenopausal and older/postmenopausal males and females

	Young/Premenopausal		Older/Postmenopausal		Effects, p(partial-η^2^)		
	Male (*n* = 53)	Female (*n* = 61)	Male (*n* = 78)	Female (*n* = 70)	Age	Sex	Age_x_sex
Age, yr	39 ± 9	37 ± 10	65 ± 7	65 ± 7	<0.001 (0.73)	0.21 (0.01)	0.15 (0.01)
BMI, kg/m^2^	28.7 ± 4.8	24.7 ± 4.1*	27.1 ± 3.4	26.5 ± 4.3†	0.77 (0.00)	<0.001 (0.07)	<0.01 (0.04)
Body fat, %^a^	21.6 ± 7.2	28.3 ± 9.9	28.9 ± 9.9	33.2 ± 10.5	<0.001 (0.09)	<0.001 (0.07)	0.26 (0.00)
Antihypertensive use, *n*(%)	3 (5.7)	3 (4.9)	29 (37.2)†	24 (34.3)†	<0.001	0.55	<0.001
Statin use, *n*(%)	1 (1.9)	0 (0.0)	29 (37.2)†	11 (15.7)†*	<0.001	<0.001	<0.001
Oral contraceptive use,^b^ n(%)	—	7 (11.7)	—	0 (0.0)	<0.001	—	—

Values are means ± SD. BMI, body mass index.

a Young male *n* = 48, premenopausal female *n* = 49, older male *n* = 78, postmenopausal female *n* = 70;

b Young-female *n* = 60, old-female *n* = 70.

* *P* < 0.05 vs. within-age males;

† *P* < 0.05 vs. young within-sex.

### Study Design

Participants were instructed to arrive at least 4-h fasted and abstain from nonessential medication (i.e., medication not prescribed for chronic conditions, allergy medication, nutritional supplements, NSAIDS), caffeine, alcohol, and exercise on the day of vascular testing. Time of day (morning) was standardized for all visits. Testing was conducted during the early follicular phase for pre- (*n* = 73) and perimenopausal (*n* = 6) participants. Premenopausal participants who used oral contraceptives (*n* = 11) were tested during the placebo week. Measurement periods were not standardized for postmenopausal participants (*n* = 72). All vascular measurements described below were obtained following 10 min of quiet, supine rest.

### Measurements

#### Descriptives.

Height and weight were measured using a stadiometer and electronic scale, respectively, and used to derive body mass index (BMI). Body fat was determined using air displacement plethysmography (Bod Pod; Cosmed, Concord, CA). Serum lipoproteins (total cholesterol, low- and high-density lipoproteins), and fasting plasma glucose were assessed using a validated point-of-care device via finger stick (Cholestech, Alere Medical) following an overnight, 12-h fast and abstinence from caffeine, alcohol, and exercise (on a separate day, when necessary).

#### Arterial stiffness.

Aortic stiffness was assessed using “gold standard” carotid-femoral (cf) pulse wave velocity (cf PWV) (52). Applanation tonometery (AtCor Medical, Sydney, Australia) was used to capture carotid and femoral artery blood pressure waveforms over a 10-s epoch along with electrocardiogram (ECG) for simultaneous R wave gating. cf PWV was calculated as the transit distance between the pulse sites divided by the time delay (peak R wave from simultaneous ECG gating to the foot of the corresponding pressure waveform) between the cf waveforms. Transit distances between carotid and femoral sites were measured as straight lines via tape measure (to nearest mm with distance adjusted for the bidirectional nature of pressure propagation via subtracting the suprasternal notch – carotid distance from the suprasternal notch – femoral distance).

Common carotid artery (CCA) stiffness was measured using ultrasound (ProSound α7; Aloka, Tokyo, Japan) and on-board wall-tracing software (eTracking). The carotid artery was imaged below the carotid bulb with a 7.5- to 10.0-MHz linear-array probe. Distances from the near wall to far wall lumen-intima interface was continuously traced using eTracking to generate a distension waveform that is analogous to pressure waveforms (33). Carotid distension waveforms were calibrated to carotid systolic (SP) and diastolic pressures (DP) acquired via contralateral applanation tonometry. Carotid distension waveforms were ensemble averaged to create a representative waveform from ≥5 waveforms collected over an ∼10- to 12-s epoch. Carotid stiffness was calculated using a local single-point PWV as $PWV-\beta =\sqrt{\beta xP_{d}/2\rho }$, where β = ln(P_s_/P_d_)/[(D_s_ – D_d_)/D_d_] and where P and D correspond to pressure and diameter respectively, and s and d refer to systolic (maximum) and diastolic (minimum) values during the cardiac cycle. cf PWV was divided by carotid PWV-β to create a ratio (Ao:CCA PWV) of the artery stiffness mismatch at the aorta-carotid interface, which has been suggested to influence pulsatility defense/transmission (32).

### Cerebrovascular Pulsatility

#### Pressure pulsatility.

Brachial SP and DP were measured in duplicate on the participant’s left arm using an oscillometric device. If duplicate values differed by more than 5 mmHg, a third measurement was obtained, and the average of the two closest measurements was used for subsequent analyses. Brachial pressure was assessed in 61.3% (*n* = 173) of the sample by using an Omron BP786N (Omron Healthcare, Inc., Lake Forest, IL), with the remaining 38.7% (*n* = 109) of the sample assessed using a Panasonic EW3109 (Panasonic Electric Works, Secaucus NJ). Both devices have been validated according to the European Society of Hypertension guidelines (3, 47). Carotid pressure waveforms (obtained via tonometry during the measurement of cf PWV, described above) were ensemble averaged to determine carotid SP. Carotid waveforms were calibrated to brachial mean pressure (MP; 1/3 SP + 2/3 DP) and DP. Pulse pressure (PP) was calculated as SP – DP.

#### Blood velocity pulsatility.

Carotid blood velocity pulsatility was measured using pulsed Doppler ultrasound with an insonation angle ≤60° and sample volume manually adjusted to encompass the entire vessel. Carotid blood velocity pulsatility index (PI) was calculated using a semiautomated flow tracing software as PI = (*V*_s_ − *V*_d_)/ *MnV*, where *V*_s_ is peak systolic, *V*_d_ is diastolic, and MnV is mean velocity. All images were stored for later offline analysis.

Cerebral blood velocity pulsatility was assessed at the MCA using Transcranial Doppler (TCD; DWL Doppler Box-X; Compumedics, Germany) and a 2-MHz transcranial probe secured to the left temporal window at a depth of ∼50–65 mm. MCA PI and mean velocity were calculated over a 6-s epoch in the same manner as described above for carotid PI and via a standard algorithm implemented on the device that utilizes a fast Fourier transform. MCA pulsatility was captured as two separate 6-s epochs that were subsequently averaged. Cerebral pulsatile damping (PD) was calculated as the ratio of proximal (carotid) to distal (MCA) pulsatility: PD = CCA PI/MCA PI (57).

#### Carotid wave dynamics.

**Fig. 1.**
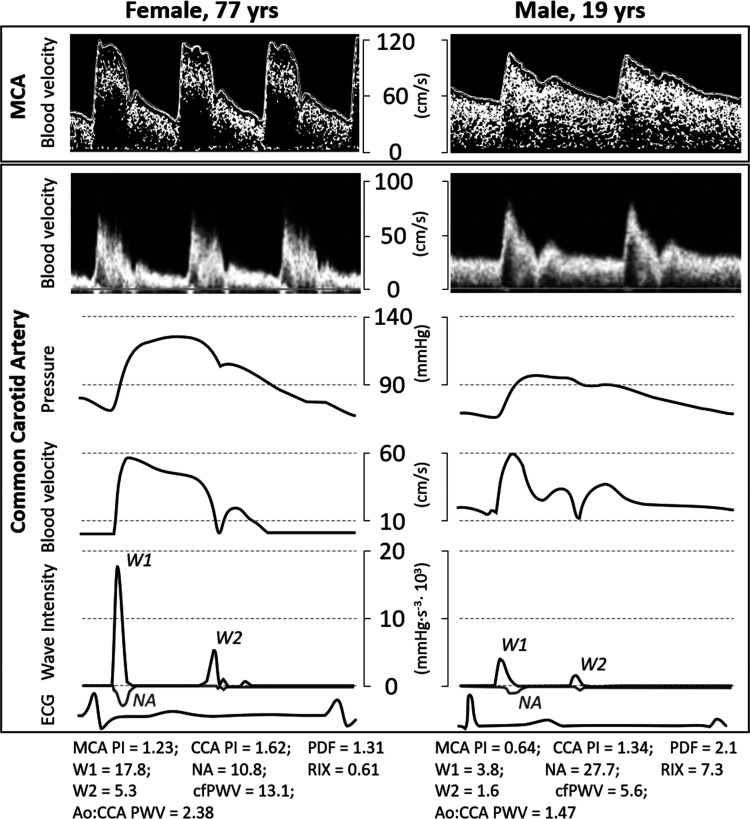
Example cerebrovascular hemodynamic waveforms contrasting an elderly female (left) compared with young male (right). Carotid pressure waveform obtained from carotid wall distension waveform and eTracking. MCA, middle cerebral artery; W1, forward wave energy; NA, negative area (reflected compression wave energy); W2, forward expansion (i.e., suction) wave energy; ECG, electrocardiogram; PI pulsatility index; PDF, pulsatile damping factor; Ao:CCA, ratio of aortic to common carotid; PWV, pulse wave velocity.

Carotid wave intensity (WI) analysis was used to gain insight into contributors to cerebrovascular pulsatile transmission/damping energetics. Carotid flow waveforms were assessed via range-gated color Doppler signals averaged along the Doppler beam over a 10- to 12-s epoch and combined with eTracking distension waveforms (described above for carotid stiffness). WI was calculated using time derivatives of blood pressure (P) and velocity (U), such that wave intensity = (dP/dt × dU/dt). WI is marked by two forward waves (W_1_, W_2_; evident by WI >0; see Fig. 1 example tracing) and one reflected wave [negative area (NA); WI <0]. The area under the dP/dt × dU/dt curve represents the energy transfer of the wave (13, 45) and is used to calculate the energy of each wave component (W_1_, W_2_, NA). W_1_ represents a forward traveling energy wave generated by the left ventricle during early systole that increases pulsatility via accelerating blood flow and increasing pressure (2); NA immediately following W_1_ is a backward traveling compression wave stemming from reflected waves from the periphery that can increase pressure pulsatility (by increasing pressure) but decrease blood velocity pulsatility (by decelerating flow) (2, 21). NA measured in the carotid has been suggested to reflect cerebrovascular tone (2). Carotid reflection index (RIx) was calculated as NA/W_1_. W_2_ is a forward traveling expansion wave generated by the initial untwist and relaxation of the left ventricle that could attenuate pulsatility by decelerating blood flow from behind (19, 34, 46). Carotid WI analysis has been shown to be reproducible (33), produces nearly identical results to wave analysis techniques using central pressure waveforms (rather than distension) (20), and has been incorporated in large-scale longitudinal investigations of brain health (6).

### Statistical Analyses

All data are reported as means ± SD, and the null hypothesis was rejected for values of *P* < 0.05. Normality of distribution for variables was assessed qualitatively using histograms and Q-Q plots as well as quantitatively using the Shapiro-Wilk test. Nonnormally distributed variables were transformed to meet normality assumptions. Descriptive characteristics and cerebrovascular hemodynamics were compared between young/premenopausal and older/postmenopausal males and females by using 2 × 2 ANOVA for continuous variables and χ^2^ tests for categorical data. Post hoc comparisons were made with a Bonferroni correction factor when a significant age-by-sex interaction was detected. Linear correlations used to examine basic relationships between age, BMI, and cerebrovascular measures are reported in the Supplemental Results.

Vascular contributors to cerebral pulsatility (MCA PI) and cerebral (MCA) pulsatile damping were selected based on hemodynamic relationships drawn from the literature (see Supplemental Table S1) and tested via multiple regression and the enter method. All models included antihypertensive and statin use as covariates. Vascular contributors to MCA PI and MCA pulsatile damping were examined using three models. Model 1 simultaneously adjusted for age [age was squared for the MCA PI model owing to nonlinear association between MCA PI and age (48)], BMI, and sex (dummy coded as 0 and 1 for male and female, respectively). Model 2 included measures of vascular structure that could impact both pulsatility and damping (aortic/carotid PWV, carotid diameter). Model 3 included hemodynamic contributors specific to 1) MCA PI [carotid PP and forward wave energy (W_1_)] or 2) MCA damping factor (carotid RIx and W_2_). All analyses were performed using Statistical Package for the Social Sciences (SPSS, version 25; IBM, Chicago IL).

## RESULTS

The original data set contained 282 participants (*n* = 131 male, *n* = 151 female). To provide a more detailed analysis of the effect of menopause within the context of aging, females who were perimenopausal (*n* = 6) or on hormone replacement therapy (*n* = 2) were excluded from the analyses. Females were divided into pre- (i.e., young) versus postmenopausal (i.e., older), and males were divided into young (18–52 yr based on the premenopausal age range) versus older (>52 yr). To eliminate an ∼5-yr mean age difference within the young males and premenopausal females (owing to more young females within the sample), 12 young premenopausal females were excluded. As such, data in the paper are presented on 262 adults (*n* = 131 males; *n* = 131 females). Descriptive characteristics for the full sample (*n* = 282) are reported in the Supplemental Results.

### Subject Characteristics and Cerebrovascular Hemodynamics

Table 1 displays participant characteristics by age (young/premenopausal versus older/postmenopausal) and sex. Age was greater in the older/postmenopausal versus young/premenopausal group (*P* < 0.05). BMI was lower among premenopausal females compared with young males and postmenopausal females (*P* < 0.05). Percent body fat was lower among males compared with females and increased from young/premenopausal to older/postmenopausal groups (*P* < 0.05). Antihypertensive use was significantly greater among older/postmenopausal compared with young/premenopausal participants and did not differ by sex. Statin use was greater in the older/postmenopausal versus young/premenopausal group, with postmenopausal females reporting less statin use than their older male counterparts (*P* < 0.05). Oral contraceptive use was greater among premenopausal versus postmenopausal females (*P* < 0.05).

Blood pressure and aortic stiffness by age (young/premenopausal vs. older/postmenopausal) and sex are presented in Table 2. Brachial diastolic pressure, aortic stiffness (cf PWV) and the ratio of aortic stiffness to carotid stiffness increased with age (main effect of age, *P* < 0.05), and aortic stiffness was higher among males than females (main effect of sex, *P* < 0.05). Age-by-sex interactions were observed for brachial systolic and mean pressure, both of which were greater among older/postmenopausal females compared with young/premenopausal females and greater among young males compared with premenopausal females (*P* < 0.05).

**Table 2.** Brachial blood pressure and aortic stiffness in young/premenopausal and older/postmenopausal males and females

	Young/Premenopausal		Older/Postmenopausal		Effects, p(partial-η^2^)		
	Male (*n* = 53)	Female (*n* = 61)	Male (*n* = 78)	Female (*n* = 70)	Age	Sex	Age_x_sex
Brachial artery							
Systolic pressure, mmHg	123 ± 10	115 ± 13*	124 ± 11	126 ± 15†	<0.001 (0.06)	0.01 (0.03)	<0.01 (0.04)
Diastolic pressure, mmHg	76 ± 8	75 ± 8	78 ± 8	79 ± 8	<0.001 (0.05)	0.61 (0.00)	0.45 (0.00)
Mean pressure, mmHg	91 ± 8	88 ± 9*	94 ± 8	94 ± 9†	<0.001 (0.06)	0.12 (0.01)	0.047 (0.02)
Aorta							
cf PWV, m/s	6.8 ± 1.2	6.4 ± 1.2*	9.3 ± 2.1†	8.8 ± 2.0†	<0.001 (0.36)	0.03 (0.02)	0.73 (0.00)
Ao:CCA PWV	1.32 ± 0.26	1.41 ± 0.29	1.54 ± 0.45	1.46 ± 0.38	0.01 (0.02)	0.70 (0.00)	0.054 (0.01)

Values are means ± SD. CCA, common carotid artery; PWV, pulse wave velocity; cf, carotid-femoral.

* *P* < 0.05 vs. within-age males;

† *P* < 0.05 vs. young within-sex.

Cerebrovascular hemodynamics by age (young/premenopausal versus older/postmenopausal) and sex are presented in Table 3. Carotid diameter, IMT, and MCA pulsatility increased, whereas carotid pulsatility index, reflected wave energy (NA), reflection index (RIx), MCA mean velocity, and MCA pulsatile damping decreased with age (main effect of age, *P* < 0.05), Carotid diameter, pulsatility index, and MCA pulsatile damping were lower among females compared with males (main effect of sex, *P* < 0.05). Age-by-sex interactions were detected for carotid systolic and pulse pressure, forward wave energy (W1), “suction” wave energy (W2), carotid stiffness (PWV-β), and MCA mean velocity. Carotid systolic and pulse pressure were lower among premenopausal females compared with young males and older/postmenopausal females (*P* < 0.05). Carotid W1 and W2 were lower among premenopausal females compared with young males (*P* < 0.05). Carotid stiffness was lower among premenopausal females compared with young males and postmenopausal females and among young males compared with older males (*P* < 0.05). MCA mean velocity was greater among premenopausal females compared with young males and was lower among older/postmenopausal males and females compared with their young/premenopausal counterparts (*P* < 0.05). Figure 1 displays individual cerebrovascular hemodynamics (wave intensity, cerebrovascular pulsatility) for an older female and a young male to contrast the effects of age and sex on outcomes of interest.

**Table 3.** Cerebrovascular hemodynamics in young/premenopausal and older/postmenopausal males and females

	Young/Premenopausal		Older/Postmenopausal		Effects, p(partial-η^2^)		
	Male (*n* = 53)	Female (*n* = 61)	Male (*n* = 78)	Female (*n* = 70)	Age	Sex	Age_×_sex
Common carotid artery							
Diameter, mm	5.73 ± 0.52	5.10 ± 0.42	5.75 ± 0.55	5.36 ± 0.51	0.03 (0.02)	<0.001 (0.20)	0.053 (0.01)
IMT, mm	0.48 ± 0.12	0.49 ± 0.12	0.70 ± 0.13	0.66 ± 0.10	<0.001 (0.40)	0.27 (0.01)	0.07 (0.01)
Pulse pressure, mmHg	38 ± 7	32 ± 8*	37 ± 7	38 ± 10†	0.01 (0.02)	<0.01 (0.04)	<0.001 (0.05)
Pulsatility index	1.66 ± 0.34	1.39 ± 0.27	1.47 ± 0.26	1.26 ± 0.21	<0.001 (0.08)	<0.001 (0.17)	0.70 (0.00)
W_1_, mmHg/m/s^3^	7.3 ± 3.2	5.3 ± 2.2*	6.7 ± 3.0	6.2 ± 3.2	0.79 (0.00)	<0.001 (0.05)	0.03 (0.02)
Negative area, mmHg/m/s^2^	32.9 ± 19.1	28.4 ± 16.3	26.3 ± 15.8	23.5 ± 14.0	0.001 (0.05)	0.054 (0.01)	0.90 (0.00)
RIx	4.8 ± 2.1	5.8 ± 2.8	4.3 ± 2.2	4.2 ± 2.2	<0.001 (0.05)	0.30 (0.00)	0.11 (0.01)
W_2_, mmHg/m/s^3^	1.6 ± 1.0	1.5 ± 0.9*	1.8 ± 0.8	1.7 ± 1.1	0.79 (0.00)	<0.001 (0.05)	0.03 (0.02)
Systolic pressure, mmHg	114 ± 10	106 ± 13*	116 ± 11	117 ± 14†	<0.001 (0.06)	0.01 (0.02)	<0.01 (0.04)
PWV-β, m/s	5.3 ± 1.0	4.6 ± 1.0*	6.2 ± 1.3†	6.2 ± 1.2†	<0.001 (0.26)	<0.01 (0.03)	<0.01 (0.03)
Middle cerebral artery							
Pulsatility index	0.73 ± 0.12	0.73 ± 0.08	0.82 ± 0.12	0.86 ± 0.14	<0.001 (0.18)	0.17 (0.01)	0.29 (0.00)
Mean velocity, cm/s	64 ± 11	74 ± 13*	55 ± 13†	57 ± 15†	<0.001 (0.20)	<0.001 (0.04)	0.02 (0.02)
Pulsatile damping	2.29 ± 0.41	1.92 ± 0.39	1.81 ± 0.32	1.47 ± 0.23	<0.001 (0.32)	<0.001 (0.22)	0.63 (0.00)

Values are means ± SD. CCA, common carotid artery; RIx, reflection index; PWV, pulse wave velocity; cf, carotid-femoral.

* *P* < 0.05 vs. within-age males;

† *P* < 0.05 vs. young within-sex.

**Fig. 2.**
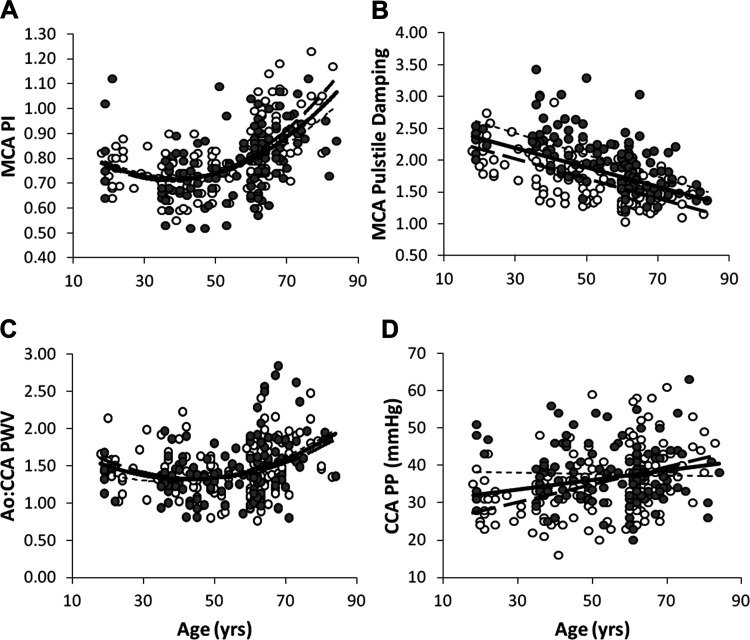
Associations between age and cerebral pulsatility (*A*), cerebral pulsatile damping (*B*), large artery stiffening (*C*), and carotid pulse pressure (*D*) (*n* = 262). *A* and *C* use quadratic lines of best fit; *B* and *D* use linear lines of best fit. MCA, middle cerebral artery; PI, pulsatility index; Ao:CCA PWV, ratio of aortic to carotid pulse wave velocity; CCA, common carotid artery; PP, pulse pressure Filled (gray) circles represent males (*n* = 131); open circles represent females (*n* = 131). Association trendlines: dashed, male; dotted, female; solid, combined sample. A: combined r = 0.55, male r = 0.47, female r = 0.64; B: combined r = −0.54, male r = −0.58, female r = −0.67; C: combined r = 0.34, male r = 0.38, female r = 0.31; D: combined r = 0.24, male r = −0.03, female r = 0.42.

Age was linearly associated with all major cerebrovascular hemodynamic measures except W_1_ and W_2_ (Supplemental Table S4). Figure 2, *A*–*D* displays associations between age and MCA pulsatility, MCA pulsatile damping, large artery stiffening (aortic:carotid PWV), and carotid pulse pressure.

### Vascular Contributors to Cerebral Pulsatility

Model 1 (age^2^, BMI, sex) from our multiple regression analyses indicated that only age (inverse) and age^2^ (positive) were significantly related to MCA pulsatility (PI; R^2^ = 0.25; Table 4). Of the structural factors included in model 2, aortic:carotid stiffness ratio (*P* = 0.08) but not carotid diameter or IMT, significantly predicted MCA PI (R^2^ = 0.26). Model 3 significantly increased prediction of MCA PI (R^2^ = 0.44), with both carotid PP and forward wave energy (W_1_) being positively related to MCA PI (*P* < 0.05). Age^2^ and aortic:carotid stiffness ratio remained significant, and sex emerged as additional significant contributors to MCA PI after accounting for carotid PP and W_1_. Antihypertensive and statin use were not predictive of MCA PI in fully adjusted models. Contributors to MCA PI identified by multiple regression remained the same in the full data set (*n* = 282).

**Table 4.** Vascular predictors of intracranial (MCA) pulsatility in adults 18–85 yr of age (*n* = 262)

	Model 1		Model 2		Model 3	
	B (95%CI)	β	B (95%CI)	β	B (95%CI)	β
Age^2^	**0.00 (0.00,0.00)**	0.45 (0.33,0.57)**	**0.00 (0.00,0.00)**	0.44 (0.28,0.59)**	**0.00 (0.00,0.00)**	0.38 (0.25,0.52)**
BMI	−0.00 (−0.01,0.00)	−0.05 (−0.16,0.06)	−0.00 (−0.01,0.00)	−0.06 (−0.16,0.06)	−0.00 (−0.01,0.00)	−0.07 (−0.17,0.03)
Female sex	0.02 (−0.01,0.05)	0.09	0.02 (−0.01,0.05)	0.07	**0.03 (0.01,0.06)**	0.13*
Ao:CCA PWV			**0.05 (0.01,0.09)**	0.15 (0.04,0.26)**	**0.06 (0.03,0.10)**	0.18 (0.08,0.27)**
CCA diameter			−0.01 (−0.03,0.02)	−0.03 (−0.15,0.10)	−0.02 (−0.05,0.01)	−0.09 (−0.19,0.02)
CCA IMT			−0.02 (−0.16,0.11)	−0.03 (−0.18,0.13)	−0.03 (−0.15,0.08)	−0.04 (−0.17,0.10)
CCA PP					**0.00 (0.00,0.01)**	0.26 (0.15,0.38)**
CCA W_1_					**0.01 (0.01,0.02)**	0.25 (0.14,0.36)**
Adjusted R^2^	**0.25**		**0.26**		**0.44**	
ΔR^2^	**0.25**		0.01		**0.18**	
F statistic	**18.19**		**12.45**		**21.44**	

Model adjusted for antihypertensive and statin medication use. BMI, body mass index; Ao:CCA PWV, ratio of aortic to carotid pulse wave velocity; CCA, common carotid artery; IMT, intima-media thickness; PP, pulse pressure; β 95% confidence interval (CI) not calculated for noncontinuous variables. Bold highlights significant effect, *P* < 0.05.

* *P* < 0.05;

** *P* < 0.01.

### Vascular Contributors to Cerebral Pulsatile Damping

Model 1 (R^2^ = 0.47) revealed that age and sex were significant negative contributors to MCA pulsatile damping, with older age and female sex being associated with lower (worse) pulsatile damping (Table 5). Aortic:carotid stiffness ratio and carotid diameter were not related to MCA pulsatile damping and did not alter the model’s prediction capacity (R^2^ = 0.47). In model 3, carotid wave reflection index (RIx), but not carotid suction wave energy, emerged as a significant contributor to pulsatile damping (R^2^ = 0.51). Significant contributions of age and sex persisted with each additional model. Antihypertensive and statin use were not predictive of MCA pulsatile damping in fully adjusted models. Contributors to MCA pulsatile damping identified by multiple regression remained the same in the full data set (*n* = 282).

**Table 5.** Vascular predictors of intracranial (MCA) pulsatile dampening in adults 18–85 yr of age (*n* = 262)

	Model 1		Model 2		Model 3	
	B (95%CI)	Β	B (95%CI)	β	B (95%CI)	β
Age	**−0.02 (−0.02,−0.01)**	−0.55 (−0.65,−0.45)**	**−0.02 (−0.02,−0.01)**	−0.56 (−0.66,−0.46)**	**−0.01 (−0.02,−0.01)**	−0.49 (−0.59,−0.39)**
BMI	0.00 (−0.01,0.01)	0.03 (−0.06,0.12)	0.00 (−0.01,0.01)	−0.02 (−0.08,0.11)	0.00 (−0.01,0.01)	0.04 (−0.05,0.13)
Sex	**−0.37 (−0.46,−0.29)**	−0.43**	**−0.36 (−0.45,−0.27)**	−0.41**	**−0.37 (−0.45,−0.28)**	−0.42**
Ao:CCA PWV			0.02 (−0.08,0.13)	0.02 (−0.07,0.11)	−0.02 (−0.12,0.09)	−0.02 (−0.11,0.07)
CCA diameter			0.04 (−0.04,0.12)	0.05 (−0.06,0.15)	0.03 (−0.04,0.11)	0.05 (−0.06,0.15)
CCA RIx					**0.04 (0.03,0.06)**	0.23 (0.14,0.33)**
CCA W_2_					−0.01 (−0.05,0.03)	−0.03 (−0.11,0.06)
Adjusted R^2^	**0.47**		**0.47**		**0.51**	
∆R^2^	**0.47**		0.00		**0.04**	
F statistic	**46.83**		**33.48**		**31.26**	

Model adjusted for antihypertensive and statin medication use. BMI, body mass index; Ao:CCA PWV, ratio of aortic to carotid pulse wave velocity; CCA, common carotid artery; RIx, reflection index. β 95% CI not calculated for non-continuous variables.

Bold highlights significant effect, *P* < 0.05.

** *P* < 0.01.

## DISCUSSION

This study sought to identify vascular contributors to cerebral pulsatility and pulsatile damping. To our knowledge, this is the first study to examine vascular contributors to pulsatile damping, an emerging concept in cerebrovascular hemodynamics. Using multiple regression we identified 1) greater aortic compared with carotid stiffening, carotid pulse pressure, and forward wave energy as significant vascular-hemodynamic contributors to MCA pulsatility; 2) carotid wave reflection as a significant vascular-hemodynamic contributor to MCA pulsatile damping; and 3) female sex as a contributor to greater cerebral pulsatility and reduced pulsatile damping in adults across the lifespan. These data suggest that wave transmission/reflection dynamics may play a role in determining cerebral pulsatility and damping and that there may be sex differences in the ability of the cerebrovasculature to defend the brain from pulsatile hemodynamics.

Our data indicate that age is one of the strongest contributors to cerebral pulsatility at the level of the MCA. Although there may be an initial, modest reduction in MCA PI in young adulthood to middle-age, there is a drastic increase in cerebral pulsatility following middle-age. This effect of aging on cerebral pulsatility has been established previously (48, 56) and appears partially related to changes in central hemodynamics (48). Indeed, our data and others’ implicate disproportionate aortic compared with carotid stiffening, central pulse pressure, and forward wave energy as major contributors to the transmission of pulsatility into cerebral vessels (27, 30, 31, 48). Data indicate the aorta stiffens with aging more so than downstream arteries (30). Disproportionate age-related increases in aortic stiffness compared with peripheral stiffness results in 1) increased forward wave energy that cannot be effectively buffered by a stiffened aorta, thereby increasing pulsatile pressure (27), and 2) a matching of the aortic:carotid stiffness gradient, reducing wave reflection and increasing transmission of forward wave energy downstream (30). In this manner, age-related changes in aortic stiffness amplify wave transmission while attenuating wave reflection (30), ultimately increasing cerebral pulsatility (10, 27, 31). Forward wave energy may be an important hemodynamic mediator of the effect of large artery stiffening on pulsatility and the brain, as greater forward wave energy in midlife is predictive of later-life cognitive decline (6). Pressure pulsatility, and thus cerebral pulsatility (27, 48), may also be driven by smaller vessel diameters (11, 29, 50). Although this has been observed at the level of the aorta (11, 29, 50), we did not identify a significant effect of carotid diameter on cerebral pulsatility. This suggests that the effect may be dependent on assessment of aortic diameter, or the modest effect of smaller carotid diameter on pulsatility is primarily explained by carotid pulse pressure and forward wave energy. Wall thickness may additionally contribute to changes in wall stiffness and thus pulsatility; however, we noted no significant effect of carotid intima-media thickness when entered into our pulsatility regression model. Cumulatively, our data corroborate the importance of age-related increases and aortic stiffness and forward wave energy transmission, which increase pressure pulsatility and ultimately contributes to cerebral pulsatility at the level of the MCA.

Age was significantly and inversely associated with pulsatile damping, similar to previous work by Zarrinkoob et al. (2016), suggesting that pulsatile damping decreases with age (57). Our data add to the previous literature by including 1) a larger sample of generally healthy adults with age (18–85 yr) as a continuous rather than a dichotomized factor and 2) individuals using antihypertensive or statin medication. As such, pulsatile damping decreases with age in healthy adults and in those undergoing pharmaceutical treatment for selected cardiovascular risk factors. Of note, the damping values of Zarrinkoob et al. (57) were generally lower than those in our sample and likely reflect differences in the upstream measurement site [internal (ICA) versus common carotid] and imaging methodology (MRI versus Doppler). Damping values calculated from the ICA would be expected to be lower, since pulsatility is greater in the common carotid and the common-internal carotid interface is a major damping site (14). The hemodynamic mechanisms contributing to pulsatile damping are discussed further below.

Our data indicate that carotid wave reflection (RIx), but not aortic:carotid stiffness ratio, carotid diameter or the forward traveling “suction” wave (W_2_), is a prominent vascular contributor to cerebral pulsatile damping in the MCA. Wave reflections, although often portrayed in a negative light (10), reduce blood flow pulsatility without altering mean flow, thereby helping attenuate blood flow pulsatility en route to sensitive end-organs (17, 21, 31). Our findings are consistent with the notion that wave reflections at the carotid-cerebral interface protect the brain from pulsatile energy (27). Carotid wave reflections may stem from the mismatch between carotid and downstream cerebral artery stiffness/diameter (27) or acute changes in cerebral tone (2). Our data suggest, however, that carotid wave reflection may have a more direct effect on cerebral pulsatile damping than the mismatch between aortic-carotid stiffness (aortic:carotid PWV) per se. Whether wave reflections generated by the stiffness mismatch at the aorta-carotid interface (i.e., upstream of the carotid) additionally contributes to cerebral pulsatile damping is physiologically logical (32) but remains untested. Our current data suggest that carotid wave reflections are a prominent vascular contributor to cerebral pulsatile damping at the level of the MCA.

Cerebral pulsatile damping was lower in females compared with males, with mean differences observed between the sexes and a significant effect of sex in regression models predicting pulsatile damping. The vascular origins of this sex-difference in pulsatile damping is unclear, but it may contribute to the small effect of female sex on greater cerebral pulsatility noted herein in the fully adjusted pulsatility model, which may be driven by larger differences in older-age males versus postmenopausal females. Although women had significantly lower forward wave energy compared with men on average, relative carotid wave reflection (expressed as RIx) was not statistically different between the sexes. This suggests that sex differences in pulsatile damping may stem from vascular factors not included in this analysis such as cerebrovascular structure/anatomy. Males appear to have greater mismatch between distal-to-proximal diameters (i.e., internal versus common carotid diameters) (41), which could support pulsatile damping by increasing reflection (5) of the larger forward wave energy among males compared with females. Moreover, tortuous portions of the cerebrovasculature (carotid siphon, Atlas slope) are important mechanisms of pulsatile damping (39, 40) that could be subject to sex differences, although this has not been directly tested. Finally, sex differences in pulsatile damping may be exaggerated by lower body wave reflections. Females exhibit greater aortic wave reflections from the lower body (25, 30), which can transition to forward (i.e., downstream) traveling energy waves at the aortic-carotid interface, altering carotid blood flow (16) and cerebral pulsatility (32). Ultimately, additional work is necessary to identify the mechanisms underlying lower cerebral pulsatile damping observed among females across the lifespan.

### Implications for Sex Differences in Brain Aging

Females appear to experience greater pulsatile burden with aging compared with their male counterparts. Cerebral pulsatility (48, 56) and pressure pulsatility (28, 43, 48) are greater in females than in males, particularly at older age, which may have implications for sex differences in brain health. Indeed, excessive cerebral pulsatility appears to damage brain structures (8) and thus contribute to the pathogenesis of cerebrovascular and cognitive disease (35, 54, 55). Sex differences in vascular contributors to brain health may play a role in observations of more severe cerebrovascular disease (42, 48) and cognitive deterioration (23) and greater lifetime risk of stroke (4) and Alzheimer’s disease in females compared with males (23). The primary vascular contributor to observed sex differences in pulsatile burden is unclear, as sex differences in artery stiffness with aging have shown mixed findings (1, 25, 48). Growing evidence suggests that the menopausal transition may be a crucial window of large artery stiffening (24, 38) and thereby influence cerebral pulsatile hemodynamics. These age-related changes in large artery stiffness and concomitant pulsatile transmission may be exaggerated by the larger age-related reductions in mean velocity (mathematical denominator in the calculation of PI) that we observed among females. Ultimately, our data indicate a potential role for attenuated pulsatile damping in females, potentially leaving the brain vulnerable to the accumulation of pulsatility-mediated damage that accompanies age/menopause-related large artery stiffening and reductions in mean velocity.

### Limitations and Considerations

Two different brachial blood pressure cuffs were used to assess blood pressure in this analysis. Both devices were shown to have acceptable agreement in blood pressure values as part of a separate, comparison substudy (see Supplemental content) with a mean difference between devices of 3 mmHg for mean pressure. Accounting for 1) blood pressure device, or 2)device-adjusted mean pressure (accounting for the 3-mmHg difference between devices) in the regression models did not alter the vascular contributors to MCA pulsatility or pulsatile damping. A larger proportion of older males reported statin use compared with postmenopausal females. Adjusting for statin-use within the older adults only, revealed that older males had significantly lower MCA PI than older females (exploratory analysis not shown). As such, statins may exert some influence on cerebral pulsatility; however, additional work is needed to directly examine this question. Future studies should calculate pulsatile damping ratios with additional extra- and intracranial arteries (common/internal carotid, anterior/middle cerebral arteries) to more comprehensively assess this novel aspect of cerebrovascular hemodynamics.

Although a growing body of literature indicates that contributors to pulsatile transmission are associated with brain health and cognitive function, further research is necessary to examine whether pulsatile damping is associated with markers of brain health and function in aging males and females. Our confirmatory findings regarding cerebral pulsatility indicate that the vascular associations detected within our sample are in-line with the literature. This strengthens and lends credence to our novel observations with respect to cerebral pulsatile damping and its contributors and suggests these may not be aberrant relations.

In summary, our data suggest that cerebral pulsatility at the level of the MCA increases with age, female sex, exaggerated aortic compared with carotid stiffening, carotid pulse pressure, and forward wave energy. We are among the first to examine vascular contributors to pulsatile damping in this setting. Pulsatile damping in the MCA decreased with age, female sex, and lower carotid wave reflections. We are the first to document decreased cerebral pulsatile damping among females compared with males, which may contribute to greater pulsatile burden among females across the lifespan. Future research is necessary to identify whether pulsatile damping plays a role in sex-differences in brain health with aging.

## GRANTS

These data were funded, in part, by American College of Sports Medicine Foundation Research Grant (W. K. Lefferts), American Heart Association Predoctoral Fellowship (16PRE31220031; W. K. Lefferts), Syracuse University Sydney Young Research Award (J. A. Augustine), Syracuse University School of Education Creative Grant Award (J. A. Augustine), Dairy Research Institute/Dairy Management Inc. (Grant 1154; K. S. Heffernan), and National Institute on Minority Health and Health Disparities (R03 MD-011306-02; K. S. Heffernan). W. K. Lefferts was supported by the National Heart, Lung, And Blood Institute of the NIH under Award Number T32 HL-134634 throughout his work on this manuscript.

## DISCLAIMERS

The content is solely the responsibility of the authors and does not necessarily represent the official views of the NIH.

## DISCLOSURES

No conflicts of interest, financial or otherwise, are declared by the authors.

## AUTHOR CONTRIBUTIONS

W.K.L. conceived and designed research; W.K.L., J.P.D., J.A.A., A.P.K., and K.S.H. performed experiments; W.K.L., J.P.D., J.A.A., A.P.K., and K.S.H. analyzed data; W.K.L., J.P.D., and K.S.H. interpreted results of experiments; W.K.L. and J.P.D. prepared figures; W.K.L. drafted manuscript; W.K.L., J.P.D., J.A.A., A.P.K., and K.S.H. edited and revised manuscript; W.K.L., J.P.D., J.A.A., A.P.K., and K.S.H. approved final version of manuscript thrroughout his work on this manuscript.

## References

[1] AlGhatrif M, Strait JB, Morrell CH, Canepa M, Wright J, Elango P, Scuteri A, Najjar SS, Ferrucci L, Lakatta EG Longitudinal trajectories of arterial stiffness and the role of blood pressure: the Baltimore Longitudinal Study of Aging. Hypertension 62: 934–941, 2013. doi:10.1161/HYPERTENSIONAHA.113.01445. 24001897 PMC3880832

[2] Bleasdale RA, Mumford CE, Campbell RI, Fraser AG, Jones CJ, Frenneaux MP Wave intensity analysis from the common carotid artery: a new noninvasive index of cerebral vasomotor tone. Heart Vessels 18: 202–206, 2003. doi:10.1007/s00380-003-0711-2. 14520489

[3] Bonso E, Dorigatti F, Palatini P Validation of Panasonic EW3106 and EW3109 devices for blood pressure measurement according to the International Protocol. Blood Press Monit 15: 55–58, 2010. doi:10.1097/MBP.0b013e32833531b3. 20071978

[4] Bushnell C, McCullough LD, Awad IA, Chireau MV, Fedder WN, Furie KL, Howard VJ, Lichtman JH, Lisabeth LD, Piña IL, Reeves MJ, Rexrode KM, Saposnik G, Singh V, Towfighi A, Vaccarino V, Walters MR; American Heart Association Stroke Council; Council on Cardiovascular and Stroke Nursing; Council on Clinical Cardiology; Council on Epidemiology and Prevention; Council for High Blood Pressure Research Guidelines for the prevention of stroke in women: a statement for healthcare professionals from the American Heart Association/American Stroke Association. Stroke 45: 1545–1588, 2014. [Errata in *Stroke* 45: e95, 2014; and *Stroke* 45: e214, 2014] doi:10.1161/01.str.0000442009.06663.48. 24503673 PMC10152977

[5] Cecelja M, Jiang B, McNeill K, Kato B, Ritter J, Spector T, Chowienczyk P Increased wave reflection rather than central arterial stiffness is the main determinant of raised pulse pressure in women and relates to mismatch in arterial dimensions: a twin study. J Am Coll Cardiol 54: 695–703, 2009. doi:10.1016/j.jacc.2009.04.068. 19679247

[6] Chiesa ST, Masi S, Shipley MJ, Ellins EA, Fraser AG, Hughes AD, Patel RS, Khir AW, Halcox JP, Singh-Manoux A, Kivimaki M, Celermajer DS, Deanfield JE Carotid artery wave intensity in mid- to late-life predicts cognitive decline: the Whitehall II study. Eur Heart J 40: 2300–2309, 2019. doi:10.1093/eurheartj/ehz189. 30957863 PMC6642727

[7] Chung CP, Lee HY, Lin PC, Wang PN Cerebral artery pulsatility is associated with cognitive impairment and predicts dementia in individuals with subjective memory decline or mild cognitive impairment. J Alzheimers Dis 60: 625–632, 2017. doi:10.3233/JAD-170349. 28826186

[8] Cooper LL, Woodard T, Sigurdsson S, van Buchem MA, Torjesen AA, Inker LA, Aspelund T, Eiriksdottir G, Harris TB, Gudnason V, Launer LJ, Mitchell GF Cerebrovascular damage mediates relations between aortic stiffness and memory. Hypertension 67: 176–182, 2016. doi:10.1161/HYPERTENSIONAHA.115.06398. 26573713 PMC4679440

[9] de la Cruz-Cosme C, Dawid-Milner MS, Ojeda-Burgos G, Gallardo-Tur A, Segura T Doppler resistivity and cerebral small vessel disease: hemodynamic structural correlation and usefulness for the etiological classification of acute ischemic stroke. J Stroke Cerebrovasc Dis 27: 3425–3435, 2018. doi:10.1016/j.jstrokecerebrovasdis.2018.08.001. 30185397

[10] de Roos A, van der Grond J, Mitchell G, Westenberg J Magnetic resonance imaging of cardiovascular function and the brain: is dementia a cardiovascular-driven disease? Circulation 135: 2178–2195, 2017. doi:10.1161/CIRCULATIONAHA.116.021978. 28559496 PMC5475278

[11] Farasat SM, Morrell CH, Scuteri A, Ting CT, Yin FC, Spurgeon HA, Chen CH, Lakatta EG, Najjar SS Pulse pressure is inversely related to aortic root diameter implications for the pathogenesis of systolic hypertension. Hypertension 51: 196–202, 2008. doi:10.1161/HYPERTENSIONAHA.107.099515. 18158348

[12] Gorelick PB, Scuteri A, Black SE, Decarli C, Greenberg SM, Iadecola C, Launer LJ, Laurent S, Lopez OL, Nyenhuis D, Petersen RC, Schneider JA, Tzourio C, Arnett DK, Bennett DA, Chui HC, Higashida RT, Lindquist R, Nilsson PM, Roman GC, Sellke FW, Seshadri S; American Heart Association Stroke Council, Council on Epidemiology and Prevention, Council on Cardiovascular Nursing, Council on Cardiovascular Radiology and Intervention, and Council on Cardiovascular Surgery and Anesthesia Vascular contributions to cognitive impairment and dementia: a statement for healthcare professionals from the American Heart Association/American Stroke Association. Stroke 42: 2672–2713, 2011. doi:10.1161/STR.0b013e3182299496. 21778438 PMC3778669

[13] Gosling RG, King DH Arterial assessment by Doppler-shift ultrasound. Proc R Soc Med 67: 447–449, 1974. 4850636 10.1177/00359157740676P113 PMC1645777

[14] Gwilliam MN, Hoggard N, Capener D, Singh P, Marzo A, Verma PK, Wilkinson ID MR derived volumetric flow rate waveforms at locations within the common carotid, internal carotid, and basilar arteries. J Cereb Blood Flow Metab 29: 1975–1982, 2009. doi:10.1038/jcbfm.2009.176. 19756018

[15] Harlow SD, Gass M, Hall JE, Lobo R, Maki P, Rebar RW, Sherman S, Sluss PM, de Villiers TJ; STRAW + 10 Collaborative Group Executive summary of the Stages of Reproductive Aging Workshop + 10: addressing the unfinished agenda of staging reproductive aging. J Clin Endocrinol Metab 97: 1159–1168, 2012. doi:10.1210/jc.2011-3362. 22344196 PMC3319184

[16] Hashimoto J, Westerhof BE, Ito S Carotid flow augmentation, arterial aging, and cerebral white matter hyperintensities. Arterioscler Thromb Vasc Biol 38: 2843–2853, 2018. doi:10.1161/ATVBAHA.118.311873. 30571170

[17] Hughes TM, Kuller LH, Barinas-Mitchell EJ, McDade EM, Klunk WE, Cohen AD, Mathis CA, Dekosky ST, Price JC, Lopez OL Arterial stiffness and β-amyloid progression in nondemented elderly adults. JAMA Neurol 71: 562–568, 2014. doi:10.1001/jamaneurol.2014.186. 24687165 PMC4267249

[18] Iadecola C, Yaffe K, Biller J, Bratzke LC, Faraci FM, Gorelick PB, Gulati M, Kamel H, Knopman DS, Launer LJ, Saczynski JS, Seshadri S, Zeki Al Hazzouri A; American Heart Association Council on Hypertension; Council on Clinical Cardiology; Council on Cardiovascular Disease in the Young; Council on Cardiovascular and Stroke Nursing; Council on Quality of Care and Outcomes Research; and Stroke Council Impact of hypertension on cognitive function: a scientific statement from the American Heart Association. Hypertension 68: e67–e94, 2016. doi:10.1161/HYP.0000000000000053. 27977393 PMC5361411

[19] Jones CJ, Sugawara M, Kondoh Y, Uchida K, Parker KH Compression and expansion wavefront travel in canine ascending aortic flow: wave intensity analysis. Heart Vessels 16: 91–98, 2002. doi:10.1007/s003800200002. 12027238

[20] Kang J, Aghilinejad A, Pahlevan NM On the accuracy of displacement-based wave intensity analysis: Effect of vessel wall viscoelasticity and nonlinearity. PLoS One 14: e0224390, 2019. doi:10.1371/journal.pone.0224390. 31675382 PMC6824577

[21] Kondiboyina A, Smolich JJ, Cheung MMH, Westerhof BE, Mynard JP Conduit arterial wave reflection promotes pressure transmission but impedes hydraulic energy transmission to the microvasculature. Am J Physiol Heart Circ Physiol 319: H66–H75, 2020. doi:10.1152/ajpheart.00733.2019. 32442033

[22] Lau KK, Pego P, Mazzucco S, Li L, Howard DP, Küker W, Rothwell PM Age and sex-specific associations of carotid pulsatility with small vessel disease burden in transient ischemic attack and ischemic stroke. Int J Stroke 13: 832–839, 2018. doi:10.1177/1747493018784448. 29966494 PMC6424409

[23] Li R, Singh M Sex differences in cognitive impairment and Alzheimer’s disease. Front Neuroendocrinol 35: 385–403, 2014. doi:10.1016/j.yfrne.2014.01.002. 24434111 PMC4087048

[24] McEniery CM Transitioning the menopause: a stiff challenge. Arterioscler Thromb Vasc Biol 40: 850–852, 2020. doi:10.1161/ATVBAHA.120.313980. 32208997

[25] McEniery CM, Yasmin, Hall IR, Qasem A, Wilkinson IB, Cockcroft JR; ACCT Investigators Normal vascular aging: differential effects on wave reflection and aortic pulse wave velocity: the Anglo-Cardiff Collaborative Trial (ACCT). J Am Coll Cardiol 46: 1753–1760, 2005. doi:10.1016/j.jacc.2005.07.037. 16256881

[26] Mitchell GF Aortic stiffness, pressure and flow pulsatility, and target organ damage. J Appl Physiol (1985) 125: 1871–1880, 2018. doi:10.1152/japplphysiol.00108.2018. 30359540 PMC6842890

[27] Mitchell GF Arterial stiffness: insights from Framingham and Iceland. Curr Opin Nephrol Hypertens 24: 1–7, 2015. doi:10.1097/MNH.0000000000000092. 25470012

[28] Mitchell GF, Gudnason V, Launer LJ, Aspelund T, Harris TB Hemodynamics of increased pulse pressure in older women in the community-based Age, Gene/Environment Susceptibility-Reykjavik Study. Hypertension 51: 1123–1128, 2008. doi:10.1161/HYPERTENSIONAHA.107.108175. 18259005 PMC11106724

[29] Mitchell GF, Lacourcière Y, Ouellet JP, Izzo JL Jr, Neutel J, Kerwin LJ, Block AJ, Pfeffer MA Determinants of elevated pulse pressure in middle-aged and older subjects with uncomplicated systolic hypertension: the role of proximal aortic diameter and the aortic pressure-flow relationship. Circulation 108: 1592–1598, 2003. doi:10.1161/01.CIR.0000093435.04334.1F. 12975261

[30] Mitchell GF, Parise H, Benjamin EJ, Larson MG, Keyes MJ, Vita JA, Vasan RS, Levy D Changes in arterial stiffness and wave reflection with advancing age in healthy men and women: the Framingham Heart Study. Hypertension 43: 1239–1245, 2004. doi:10.1161/01.HYP.0000128420.01881.aa. 15123572

[31] Mitchell GF, van Buchem MA, Sigurdsson S, Gotal JD, Jonsdottir MK, Kjartansson Ó, Garcia M, Aspelund T, Harris TB, Gudnason V, Launer LJ Arterial stiffness, pressure and flow pulsatility and brain structure and function: the Age, Gene/Environment Susceptibility--Reykjavik Study. Brain 134: 3398–3407, 2011. doi:10.1093/brain/awr253. 22075523 PMC3212721

[32] Mynard JP, Kowalski R, Cheung MM, Smolich JJ Beyond the aorta: partial transmission of reflected waves from aortic coarctation into supra-aortic branches modulates cerebral hemodynamics and left ventricular load. Biomech Model Mechanobiol 16: 635–650, 2017. doi:10.1007/s10237-016-0842-x. 27730475

[33] Niki K, Sugawara M, Chang D, Harada A, Okada T, Sakai R, Uchida K, Tanaka R, Mumford CE A new noninvasive measurement system for wave intensity: evaluation of carotid arterial wave intensity and reproducibility. Heart Vessels 17: 12–21, 2002. doi:10.1007/s003800200037. 12434197

[34] Parker KH, Jones CJ, Dawson JR, Gibson DG What stops the flow of blood from the heart? Heart Vessels 4: 241–245, 1988. doi:10.1007/BF02058593. 3254905

[35] Pase MP, Beiser A, Himali JJ, Tsao C, Satizabal CL, Vasan RS, Seshadri S, Mitchell GF Aortic stiffness and the risk of incident mild cognitive impairment and dementia. Stroke 47: 2256–2261, 2016. doi:10.1161/STROKEAHA.116.013508. 27491735 PMC4995162

[36] Purkayastha S, Fadar O, Mehregan A, Salat DH, Moscufo N, Meier DS, Guttmann CR, Fisher ND, Lipsitz LA, Sorond FA Impaired cerebrovascular hemodynamics are associated with cerebral white matter damage. J Cereb Blood Flow Metab 34: 228–234, 2014. doi:10.1038/jcbfm.2013.180. 24129749 PMC3915198

[37] Rivera-Rivera LA, Turski P, Johnson KM, Hoffman C, Berman SE, Kilgas P, Rowley HA, Carlsson CM, Johnson SC, Wieben O 4D flow MRI for intracranial hemodynamics assessment in Alzheimer’s disease. J Cereb Blood Flow Metab 36: 1718–1730, 2016. doi:10.1177/0271678X15617171. 26661239 PMC5076787

[38] Samargandy S, Matthews KA, Brooks MM, Barinas-Mitchell E, Magnani JW, Janssen I, Hollenberg SM, El Khoudary SR Arterial stiffness accelerates within 1 year of the final menstrual period: the SWAN Heart Study. Arterioscler Thromb Vasc Biol 40: 1001–1008, 2020. doi:10.1161/ATVBAHA.119.313622. 31969013 PMC7101253

[39] Schubert T, Pansini M, Bieri O, Stippich C, Wetzel S, Schaedelin S, von Hessling A, Santini F Attenuation of blood flow pulsatility along the Atlas slope: a physiologic property of the distal vertebral artery? AJNR Am J Neuroradiol 36: 562–567, 2015. doi:10.3174/ajnr.A4148. 25395658 PMC8013071

[40] Schubert T, Santini F, Stalder AF, Bock J, Meckel S, Bonati L, Markl M, Wetzel S Dampening of blood-flow pulsatility along the carotid siphon: does form follow function? AJNR Am J Neuroradiol 32: 1107–1112, 2011. doi:10.3174/ajnr.A2426. 21474624 PMC8013150

[41] Schulz UG, Rothwell PM Sex differences in carotid bifurcation anatomy and the distribution of atherosclerotic plaque. Stroke 32: 1525–1531, 2001. doi:10.1161/01.STR.32.7.1525. 11441196

[42] Simoni M, Li L, Paul NL, Gruter BE, Schulz UG, Küker W, Rothwell PM Age- and sex-specific rates of leukoaraiosis in TIA and stroke patients: population-based study. Neurology 79: 1215–1222, 2012. doi:10.1212/WNL.0b013e31826b951e. 22955138 PMC3440447

[43] Smulyan H, Asmar RG, Rudnicki A, London GM, Safar ME Comparative effects of aging in men and women on the properties of the arterial tree. J Am Coll Cardiol 37: 1374–1380, 2001. doi:10.1016/S0735-1097(01)01166-4. 11300449

[44] Snyder HM, Corriveau RA, Craft S, Faber JE, Greenberg SM, Knopman D, Lamb BT, Montine TJ, Nedergaard M, Schaffer CB, Schneider JA, Wellington C, Wilcock DM, Zipfel GJ, Zlokovic B, Bain LJ, Bosetti F, Galis ZS, Koroshetz W, Carrillo MC Vascular contributions to cognitive impairment and dementia including Alzheimer’s disease. Alzheimers Dement 11: 710–717, 2015. doi:10.1016/j.jalz.2014.10.008. 25510382 PMC4731036

[45] Sugawara M, Niki K, Ohte N, Okada T, Harada A Clinical usefulness of wave intensity analysis. Med Biol Eng Comput 47: 197–206, 2009. doi:10.1007/s11517-008-0388-x. 18763005

[46] Sugawara M, Uchida K, Kondoh Y, Magosaki N, Niki K, Jones CJ, Sugimachi M, Sunagawa K Aortic blood momentum--the more the better for the ejecting heart in vivo? Cardiovasc Res 33: 433–446, 1997. doi:10.1016/S0008-6363(96)00241-6. 9074709

[47] Takahashi H, Yokoi T, Yoshika M Validation of the OMRON M6 Comfort (HEM-7321-E) upper arm blood pressure monitor, in oscillometry mode, for clinic use and self measurement in a general population, according to the European Society of Hypertension International Protocol revision 2010. Duplin: dablEducational Trust, http://www.dableducational.org/Publications/2014/ESH-IP2010ValidationofOmronM2016Comfort(HEM-7321-E).pdf, 2014.

[48] Tarumi T, Ayaz Khan M, Liu J, Tseng BY, Parker R, Riley J, Tinajero C, Zhang R Cerebral hemodynamics in normal aging: central artery stiffness, wave reflection, and pressure pulsatility. J Cereb Blood Flow Metab 34: 971–978, 2014. doi:10.1038/jcbfm.2014.44. 24643081 PMC4050241

[50] Torjesen AA, Sigurðsson S, Westenberg JJ, Gotal JD, Bell V, Aspelund T, Launer LJ, de Roos A, Gudnason V, Harris TB, Mitchell GF Pulse pressure relation to aortic and left ventricular structure in the Age, Gene/Environment Susceptibility (AGES)-Reykjavik Study. Hypertension 64: 756–761, 2014. doi:10.1161/HYPERTENSIONAHA.114.03870. 25024287 PMC4162768

[51] Toth P, Tarantini S, Csiszar A, Ungvari Z Functional vascular contributions to cognitive impairment and dementia: mechanisms and consequences of cerebral autoregulatory dysfunction, endothelial impairment, and neurovascular uncoupling in aging. Am J Physiol Heart Circ Physiol 312: H1–H20, 2017. doi:10.1152/ajpheart.00581.2016. 27793855 PMC5283909

[52] Townsend RR, Wilkinson IB, Schiffrin EL, Avolio AP, Chirinos JA, Cockcroft JR, Heffernan KS, Lakatta EG, McEniery CM, Mitchell GF, Najjar SS, Nichols WW, Urbina EM, Weber T; American Heart Association Council on Hypertension Recommendations for improving and standardizing vascular research on arterial stiffness: a scientific statement from the American Heart Association. Hypertension 66: 698–722, 2015. doi:10.1161/HYP.0000000000000033. 26160955 PMC4587661

[53] Wåhlin A, Ambarki K, Birgander R, Malm J, Eklund A Intracranial pulsatility is associated with regional brain volume in elderly individuals. Neurobiol Aging 35: 365–372, 2014. doi:10.1016/j.neurobiolaging.2013.08.026. 24080175

[54] Webb AJ, Simoni M, Mazzucco S, Kuker W, Schulz U, Rothwell PM Increased cerebral arterial pulsatility in patients with leukoaraiosis: arterial stiffness enhances transmission of aortic pulsatility. Stroke 43: 2631–2636, 2012. doi:10.1161/STROKEAHA.112.655837. 22923446

[55] Wohlfahrt P, Krajcoviechova A, Jozifova M, Mayer O, Vanek J, Filipovsky J, Laurent S, Cifkova R Large artery stiffness and carotid flow pulsatility in stroke survivors. J Hypertens 32: 1097–1103, 2014. doi:10.1097/HJH.0000000000000137. 24569418

[56] Yang D, Cabral D, Gaspard EN, Lipton RB, Rundek T, Derby CA Cerebral hemodynamics in the elderly: a transcranial Doppler study in the Einstein Aging Study Cohort. J Ultrasound Med 35: 1907–1914, 2016. doi:10.7863/ultra.15.10040. 27417737 PMC5500193

[57] Zarrinkoob L, Ambarki K, Wåhlin A, Birgander R, Carlberg B, Eklund A, Malm J Aging alters the dampening of pulsatile blood flow in cerebral arteries. J Cereb Blood Flow Metab 36: 1519–1527, 2016. doi:10.1177/0271678X16629486. 26823470 PMC5012521

